# Permeability and Equivalent Circuit Model of Ionically Conductive Mortar Using Electrochemical Workstation

**DOI:** 10.3390/ma13051179

**Published:** 2020-03-06

**Authors:** An Xu, Yubin Weng, Ruohong Zhao

**Affiliations:** Guangzhou University-Tamkang University Joint Research Center for Engineering Structure Disaster Prevention and Control, Guangzhou University, Guangzhou 510006, China; xuan@gzhu.edu.cn (A.X.); 2111616188@e.gzhu.edu.cn (Y.W.)

**Keywords:** electrochemical workstation, ionically conductive mortar, open-circuit potential, EIS, quasi-Randles model

## Abstract

Ionically conductive mortar is a new Portland cement-based construction material prepared by permeating electrolyte solution into porous mortar specimen. The conductive mechanism of ionically conductive mortar is the directional movement of internal free ions under external electric field. Because of the strong electrochemical properties of ionically conductive mortar, electrochemical workstation was used to test the performance of ionically conductive mortar. The open-circuit potential during the permeation process of ionically conductive mortar was tested. The results show that the change of open-circuit potential can effectively reflect the permeability rate of the samples with different porosity and electrolyte mass fraction. Besides the permeation of specimen, electrochemical workstation was also used to test the EIS (electrochemical impedance spectroscopy) of permeated specimens with different porosity, concentration of electrolyte solution, and different kinds of electrolyte solution. The quasi-Randles circuit model was then used to establish an equivalent circuit of ionically conductive mortar. Finally, the relation between parameter of circuit and the porosity or electrolyte solution was established. The test results show that solution resistance of the equivalent circuit and real resistivity of specimens is linearly correlated. This shows the equivalent circuit can effectively reflect the real resistivity of ionically conductive mortar, and the variation of electronic component parameters of equivalent circuit conforms to the conductive mechanism of ionically conductive mortar.

## 1. Introduction

Conductive concrete is a new construction material developed in 1970s. Conductive concrete can be used in deicing, snow-melting, electromagnetic shielding, etc. Traditional conductive concrete usually is manufactured by mixing conductive material, such as steel fiber [1], carbon fiber [2], graphite [3], or carbon black [4] into concrete. There are many drawbacks of the conductive concrete, such as the rusting steel fiber [5] and non-uniform dispersion of conductive materials [2]. Different from traditional conductive concrete, ionically conductive mortar is a new type of construction material. Ionically conductive mortar can be manufactured by immersing the porous cement mortar (porosity 17% ~ 35%) into the electrolyte solution for a certain period of time [6,7,8]. The conductive circuit of the traditional conductive concrete consists of conductive materials inside the concrete such as steel fiber or graphite [1,2,3,4]. Meanwhile, the conductive mechanism of ionically conductive mortar is the movement of internal free ions under external electric load. Ionically conductive mortar has excellent conductivity and electric-heating performance [6,7,8], and can be used as the internal partition wall of the building for indoor heating [9]. The mobility of ionically conductive mortar is based on the amount of moisture and free ions inside mortar, references [7,9] provide the effective method to maintain the mobility of ionically conductive mortar by supplying the electrolyte solution. Previous experimental results have shown that the conductivity of ionically conductive mortar is determined by its porosity, the concentration, and type of electrolyte solution penetrated inside the cement [6,7,8]. However, it is very difficult to establish the mathematical relationships between the resistivity of ionically conductive mortar and these parameters by using traditional methods.

Electrochemical workstation is an instrument that is widely used in the fields of biology, chemistry, and electrochemistry to measure the electrochemical characteristics of materials. The application of electrochemical workstations in traditional Portland cement began in the 1980s. McCarter [10,11] was among the earliest to perform electrochemical impedance spectroscopy (EIS) of hardened cement paste. Subsequently, some scholars [12,13] proved that the EIS characteristics of cementitious materials are related to water-cement ratio and hydration time. Based on this, the interface model and equivalent circuit of cement was developed to describe the EIS during cement hydration [14,15]. In recent years, the application of electrochemical workstations in cement area mainly focuses on the study of chloride ion expansion and reinforcement corrosion [16,17,18,19]. Electrochemical workstation also has been used to study the basic performance of cement and new construction technology, such as the hydration [20], durability [21], cement surface modification [22] etc. Especially, the microstructure and EIS of cement has been related to discover the mechanisms of cement hydration [23,24].

In this regard, the EIS method offers a promising approach for characterizing ionically conductive mortar. This method can be used to measure the ac impedance spectrum, so as to analyze the influence of material parameters (porosity, electrolyte solution concentration, type of electrolyte solution, etc.,) on its electrically properties.

To our knowledge, the EIS technology has not been applied to ionically conductive mortar. It was found there is a linear relation between the reciprocal of the square root of ion concentration and the diameter of semi-circle in high frequency area in EIS, it also found there is a inversely proportional relation between the impedance semicircle diameter and the product by porosity and average pore diameter [25,26]. 

Based on these, electrochemical workstation was adopted to test the property of ionically conductive mortar. Open-circuit potential during the permeating process of ionically conductive mortar was dated to research the influence of different porosity and electrolyte solution concentration on the permeating velocity and effects, and the general rules of the permeating process of ionically conductive mortar was summarized. Further, the EIS of ionically conductive mortar specimens with different porosity, electrolyte solutions concentrations, and different kinds of electrolyte solutions were tested by electrochemical workstation, and the equivalent circuits of specimens were developed, the relation between parameters of equivalent circuit and the porosity of the samples was established, as well as the electrolyte solution.

## 2. Specimen Preparation

The mortar specimens were prepared according to national standard GB/T17671 1999 [27], with a 1:3 mass ratio of cement to sand. The properties of materials used in preparation are shown in Table 1, Table 2 and Table 3. 

The dimensions of the specimens were 40 mm × 40 mm × 40 mm. The fabrication process is summarized as follows: (1) The quantities of cement and sand were mixed in a mixer for 1 min; (2) tap water was added into the mixture and mixed for 1.5 min; (3) air-entraining agent aluminum powder and SJ-2 were added and mixed for 1 min; (4) the mixture was cast into a mold in three times and vibrated on a vibrating table for about 5–6 sec after each casting; (5) after casting 1/3 of the paste, the electrode was immersed into the mold, and the location of electrode was adjusted after each casting; and (6) the surface of the specimen was finished with a steel trowel. 

The specimens were taken out of the mold after about 24 hours and put into a curing box of 20 °C (68oF) and 98% humidity for 28 days [28]. The specimens were dried in vacuum drying oven at 60 °C (140oF) for 8 hours and weighed after drying. 

**Figure 1:**
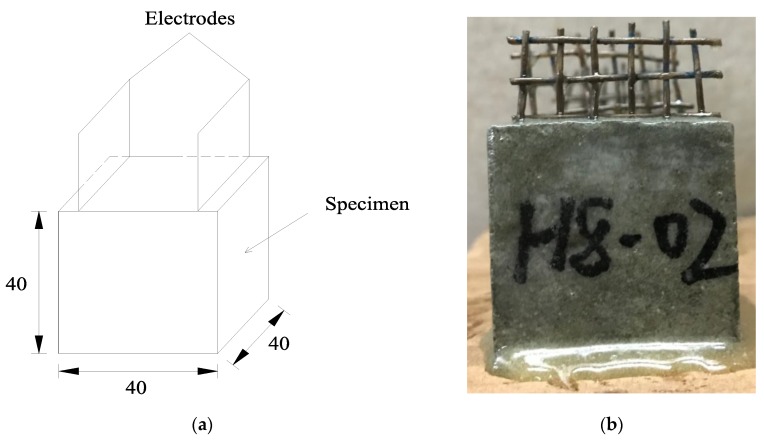
Typical ionically conductive mortar. (**a**) the dimension of specimen (**b**) photo of specimen.

There are two tests in this paper including open-circuit potential test and EIS test. The specimens used to test the open-circuit potential were prepared after curing and drying, and the codes of specimens in open-circuit potential test are shown in Table 4. The specimens used to test EIS were immersed in electrolyte solutions after curing and drying. Different solute substance was chosen according to the different experiment purpose. The codes of specimens in EIS test are shown in Table 5. All the specimens were taken out of the electrolyte solution after being immersed 96 hours to ascertain saturation of electrolyte. After immersion was complete, the mortar specimens were wiped dry and coated with a 1 mm-thick layer of epoxy resin to prevent evaporation of moisture. Figure 1 shows a typical test specimen. 

## 3. Open-Circuit Potential Test during Permeating Process 

The conductivity of ionically conductive mortar mainly depends on the number of free ions and moisture inside the mortar. Therefore, the permeation step in the preparation process is very important. The permeation efficiency depends on the porosity of the specimen and the concentration of the electrolyte solution used. The electrochemical workstation was used to test the open-circuit potential changes of samples with different porosity and electrolyte solution concentration during the permeation process. The specimen used in this section is one that has not been penetrated by electrolyte solution after curing for 28 d and drying. After drying the cement mortar specimen can be considered as an insulator because of small internal moisture. When one of the surfaces of the specimen was exposed to the electrolyte solution for the penetration test, the electrolyte solution permeated to the specimens. When the electrode inside specimen contacted with the solution, the electrode surface was dissolved by the electrolyte solution and free ions was adsorbed by the electrode because of the different free energy of the two phases of the electrode and the solution, consequently causing the potential to change on the electrode surface. The change of open-circuit potential reflects the unstable to stable process of potential between the two electrodes. 

### 3.1. Test Method

**Figure 2:**
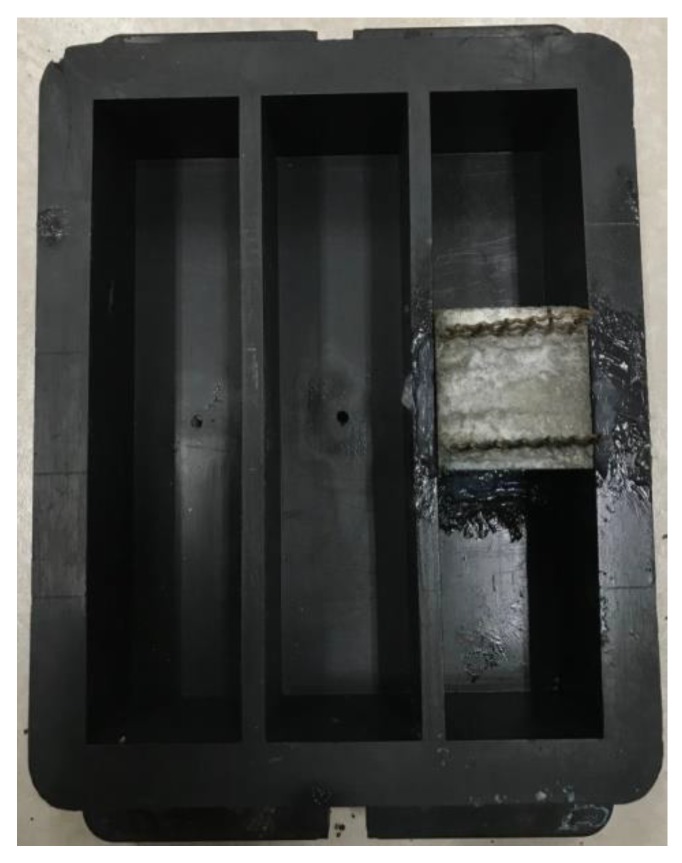
The tank used in permeating test.

**Figure 3:**
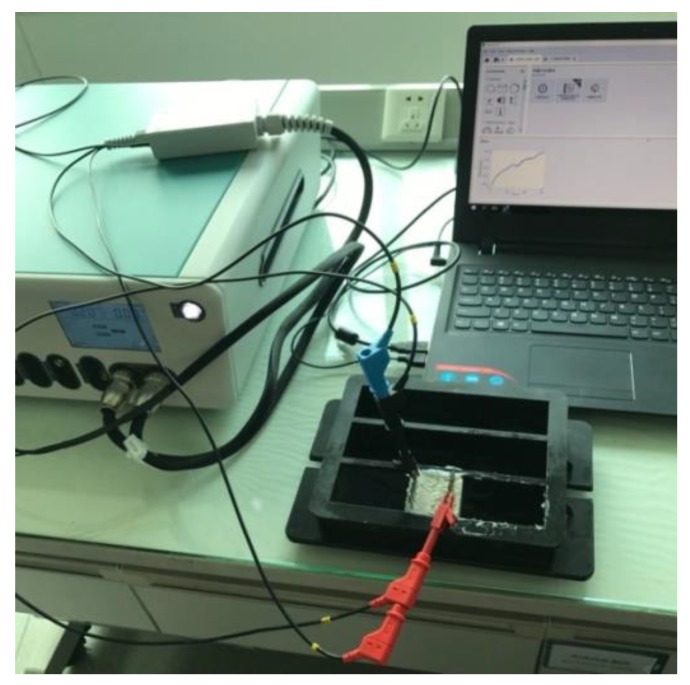
Open circuit potential test.

The electrochemical workstation used was Autolab M204 manufactured by Metrohm company. The tank used in the permeating test is shown in Figure 2. Vaseline was applied to both sides and bottom of the specimen to prevent the leaking of the electrolyte solution. The specimen was placed in the middle of the tank, and the electrode and electrochemical workstation measuring plug was firmly connected to ensure a stable state of connection. Electrolyte solution was poured in one side of the tank until the height of the electrolyte solution was equal to the specimens. The data collection of open circuit potential was then performed for a test time of 15 min at the time interval of 0.2 s as shown in Figure 3.

### 3.2. Analysis of Specimens with Different Porosity

**Figure 4:**
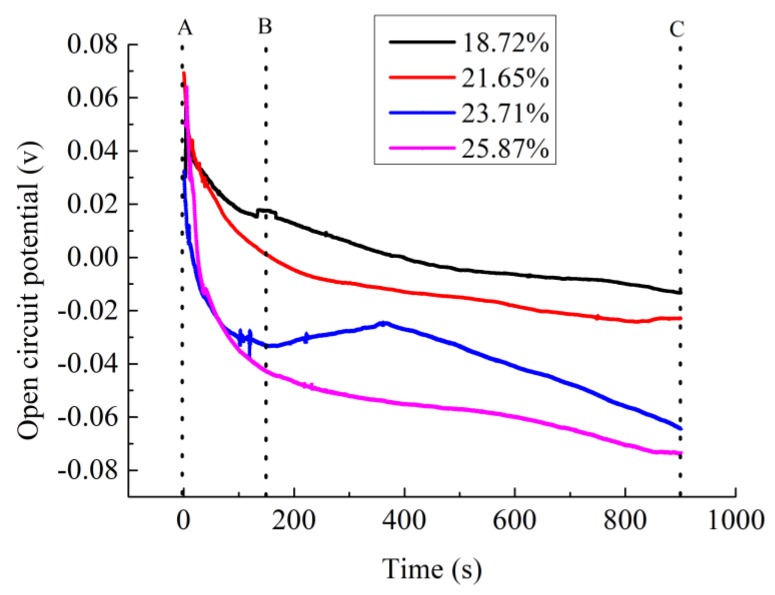
Change of open circuit potential with time of different porosity specimens.

In order to explore the rule of penetration of specimens with different porosity, four specimens made in the same period but different porosity were selected. The porosity of specimens, which was controlled by the mix ratio of air entraining agent [6], was 18.72%, 21.65%, 23.71%, and 25.87%, respectively. The electrolyte solution is CaCl_2_ with 10% mass fraction. The open circuit potential data of the above four samples was tested. The experiment results are shown in Table 6 and Figure 4.

It can be found from Figure 4 and Table 6 that the permeability rule of specimens with different porosity is similar under the same experimental conditions. The change of open-circuit potential ∆V between 0 min to 15 mins increases with the increase of porosity. The open circuit potential of specimens changed during the permeating process, it shows that originally stable status inside the specimens are destroyed with the penetration of the electrolyte solution. The variation of the open circuit potential was influenced by the amount of electrolyte solution permeated in the specimens and the number of inner free ion; the larger the open circuit potential variation, the greater the electrolyte solution permeated, and the more number of free ions. 

As showed in Figure 4, the permeating process of the four samples can be roughly divided into two stages: the rapid permeating stage (AB stage) and the slow permeating stage (BC stage). AB stage presents the beginning of the permeating process. After curing for 28 day and drying, the specimen was in a relatively stable state. This stability was broken when one side of the specimen contacts the electrolyte solution. The open circuit potential between both electrodes significantly changed in very short time shown as AB stage in Figure 4. However, after the initial drastic change, the open circuit potential curve becomes flat gradually as shown in BC stage of Figure 4. In this period, electrolyte solutions had permeated into the interior of the specimen. At this time, the electrolyte solution of the internal specimen and the external tank were relatively in balance. Thus, although the permeating process continues, the change of open circuit potential is not as large as the beginning stage.

By comparing the open circuit potential curves of specimens with different porosity, it can be found that the slope of the curve between AB stage increases with the increase of porosity, as well as the change of open circuit potential (∆V). Obviously, larger porosity of the specimens results in larger penetration of the electrolyte solution. In BC stage, the open circuit potential of specimens with small porosity (18.72% and 21.65%) is much flat than that of the specimens with large porosity (23.71% and 25.87%). The curve of the specimen with large porosity still has the obvious downward trend during the slow permeating process. It shows that the larger the porosity, the higher the stability of the electrolyte solution between internal and external specimen can get. Thus, the permeating process of large porosity specimen is longer than that with small porosity. In practical experiment, the permeating time of large porosity specimen should be extended to get the sufficient penetration. 

Table 6 also shows the real resistivity of every specimen which decreases with the increasing of porosity. This rule is completely consistent with the results of this permeating test. It can be shown that the test of open circuit potential by using electrochemical workstation can effectively reflect the permeation rule of ionically conductive mortar with different porosity.

### 3.3. Analyze the Test Result of Specimens with Different Electrolyte Solution Concentration

**Figure 5:**
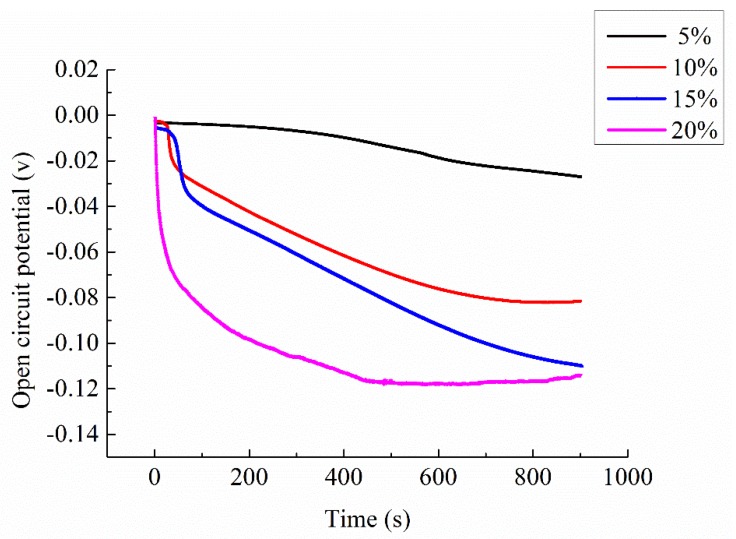
Change of open circuit potential of specimens with different mass ratio electrolyte solution.

Four specimens (porosity ranging from 18.2% to 18.5%) made in the same period and by the same formula were chosen. After the 28 days curing period, the specimens were taken out of the curing box and dried for further test. The electrolyte solution used in permeating process was CaCl_2_, and mass fractions of it were 5%, 10%, 15%, and 20%, respectively. The open circuit potential of four specimens permeated by different mass fraction electrolyte solution were tested. Test results are shown in Table 7 and Figure 5.

From Figure 5, it can be found that with the increasing of the mass fraction of electrolyte solution, the open circuit potential of the specimen decreases. Clearly, the mass ratio of electrolyte solution is an important factor that affects the permeability of the specimen.

When the mass ratio of electrolyte solution was 5%, the open-circuit potential change of the specimen was very gentle even in the rapid permeating stage. Combining the data shown in Table 7, the total open-circuit potential change ∆V of this specimen was only 0.0237, which was much lower than that of the other three groups. As mentioned above, the change of open circuit potential in the permeation process is directly related to the amount of electrolyte solution permeating into the specimen. The test result indicates that if the mass ratio of electrolyte solution is too small (5%), there would be not enough active free ions permeating into the specimen, which also means the low permeation efficiency. This is because when the concentration of electrolyte solution is low, the distance between the ions is relatively long and the distance for a single ion to pass through is relatively long. In the process of penetration, the number of ions permeating into the specimen within the same time is less than that with high mass ratio. Consequently, it caused the relatively high real resistivity of specimens as shown in Table 5. The resistivity of specimen with 5% electrolyte solution is 1.07 Ω⋅m, which is 45% larger than that of the specimens with 10% solution. 

By observing the two curves with mass ratio of 10% and 15%, it can be found that variation tendency of the two curves are relatively similar, but the value of specimen with 15% electrolyte solution is slightly lower than that with 10% mass ratio. At the end of the test, the curve of the specimen with 10% electrolyte solution begins to stabilize, while the curve of the 15% mass ratio still shows a downward tendency. This also resulted in the difference in open-circuit potential change ∆V between the two specimens at the end of the test, which was 0.0790 and 0.0944, respectively, as shown in Table 7. The real resistivity of these two specimens is 0.59 Ω⋅m and 0.44 Ω⋅m, respectively, which also decreases with the increasing of the mass ratio of the electrolyte solution.

When the mass fraction of electrolyte solution increases to 20%, it can be found that the curve declines rapidly in the rapid permeating stage. In slow permeating stage, the curve become very gentle, the curve slope approximates that of specimen with 5% electrolyte solution. This is because at the beginning of penetration, electrolyte solution with large mass ratio can provide large amounts of free ions in a short time. The amount of free ions absorbed by the surface of the two electrodes are different; the potential between the two electrodes is much larger than that of the specimen with small mass ratio electrolyte solution, consequently causing the rapid decline of open circuit potential curve. As penetration process continues, a certain amount of electrolyte solution already exists inside the mortar specimen. At this point, the viscosity of the electrolyte solution is the main factor that restricts the ion movement; the viscosity of the electrolyte solution increases with the increase of mass fraction of the solution [29]. The interaction force between the ions increases with the increasing viscosity, which causes the resistance to the free ions during the permeating process, consequently affecting the penetration efficiency of the electrolyte solution. On the other hand, in electrolyte solution, besides the interaction between ions, ions and water molecules also interact with each other, which is called hydration of ions [30]. Some polar water molecules are oriented around the ions and closely bind with ions to form hydration ions. The water molecules are bound in the solvation layer around the ions and cannot move independently, but can only move together with the ions. This reduces the number of free water molecules, which is equivalent to the increase of the actual concentration of ions. When the mass ratio of solution is relatively low, the number of water molecules is far greater than the number of ions, which means the hydration does not impact the concentration significantly. However, as the mass ratio increases, the proportion of free water molecules becomes smaller and the influence of hydration becomes larger. In fact, the movement of H^+^ and OH^−^ is faster than other ions such as Cu^2+^ or Ca^2+^ [29], thus, during the permeating process, the water permeate in the specimens before electrolyte. This also causes the concentration of electrolyte solution to become higher than the original solution, consequently enhancing the effectiveness of hydration. These are the reasons that the curve slope of the specimen with 20% electrolyte solution declines. The real resistivity of specimen with 20% electrolyte solution is 0.42 Ω⋅m which only decreases 4.5% than that of the 15% electrolyte solution. It can be predicted that the resistivity of the specimen will not decrease greatly with the further increasing of the mass ratio of the electrolyte solution. 

Combining the results of Section 3.2 and Section 3.3, open circuit potential data tested during the permeating process conforms the real resistivity of the ionically conductive mortar specimens. This shows that the open-circuit potential tested by the electrochemical workstation can effectively reflect the permeability of ionically conductive mortar influenced by the mass fraction of the electrolyte solution and the porosity of the specimen.

## 4. EIS and Equivalent Circuit of Ionically Conductive Mortar

As mentioned before, the internal pores of ionically conductive mortar are filled with electrolyte solution. Since the solid matrix of the mortar is basically an insulating material, the impedance of the ionic conductive mortar is almost completely determined by its pore structure characteristics and the electrolyte solution inside mortar. Various ions in the electrolyte solution inside the mortar specimen, together with the capillary pores, gel pores, micro-cracks, micro-pores, and the solid matrix of the mortar, constitute a complex electrochemical system [31]. We have determined the EIS of ionically conductive mortar with different porosity, concentration of electrolyte solution, and type of electrolyte solution using the electrochemical workstation. Additionally, equivalent internal circuit of ionically conductive mortar have been simulated. Based on this, the relationship between the element parameters (solution resistance, and charge transfer resistance, capacitance solid–liquid interface, etc.,) of equivalent circuit and the parameters of specimen (porosity, electrolyte solution types, electrolyte solution mass fraction) was stablished.

### 4.1. Circuit Model of Ionically Conductive Mortar

**Figure 6:**
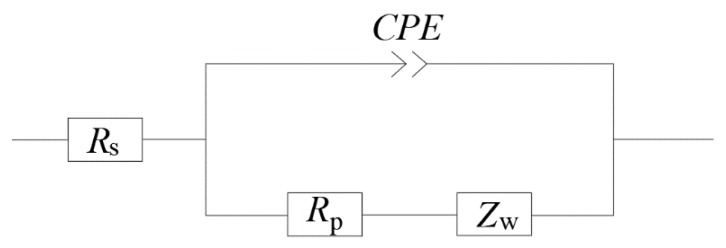
Equivalent circuit model of ionically conductive mortar.

The equivalent circuit of the electrochemical system in ionically conductive mortar can be expressed as the circuit shown in Figure 6, which is called quasi-Randles model, where,$R_{S}$ is the solution resistance; $R_{P}$ is the charge-transfer resistance, namely interface reaction resistance; $Z_{W}$ is the diffusion impedance; CPE is the capacitance in the solid–liquid interface.

An electrochemical system inside the mortar is essentially composed of two electrodes and the electrolyte between the electrodes. There are two processes proceeding synchronously: the capacitance in the system is charged and discharged with the change of electrode potential; and Faraday process when the electrode potential remains constant [32]. Therefore, in the equivalent circuit, Faraday impedance can be expressed by the series consisting of charge transfer resistor *R_ct_* and impedance $Z_{D}$ of the diffusion process. The diffusion impedance $Z_{D}$ can be divided into different types according to different electrochemical systems. The diffusion impedance of ionically conductive mortar is the semi-infinite diffusion impedance of the planar electrode, i.e. Warburg impedance [32].

In circuit model, to avoid "dispersion effects," capacitors of electrochemical system are usually replaced with phase-angle elements CPE [33]. Common electrochemical systems are composed of electrodes and electrolyte solutions, and the only factor that can cause the phase-constant angle element is the roughness of the electrode surface. However, for porous materials such as ionically conductive mortar, the internal complex pore structure can also cause the phase-constant angle element [33]. In addition, compared with the electrode roughness factor, the influence of pore structure is much greater than the former [34]. Therefore, for the electrochemical system inside the ionically conductive mortar, the influence of electrode surface roughness can be ignored.

Under electric field, parallel plate capacitor of electrochemical system is formed by directional arrangement of various ions on the solid–liquid interface, which is caused by the potential difference between the slurry solid matrix and pore solution [26]. Thus, ionically conductive mortar also has electrochemical capacitors with solid–liquid interface, and the size of the capacitance is determined by the pore structure characteristics of the mortar.

**Figure 7:**
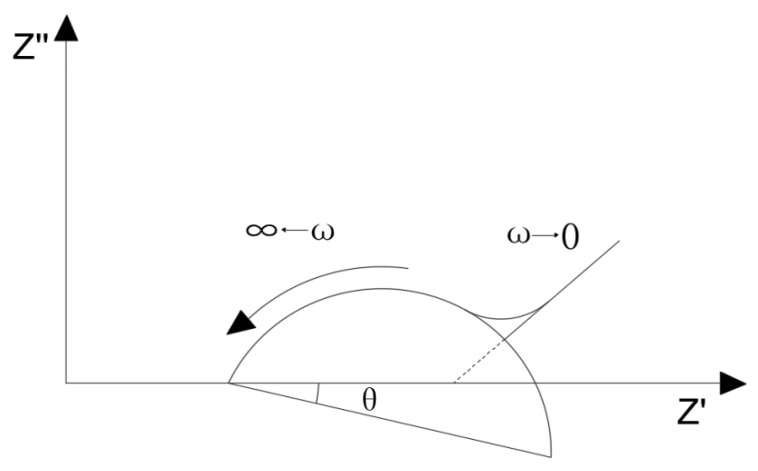
Nyquist diagram corresponding to the quasi-Randles model [25].

**Figure 8:**
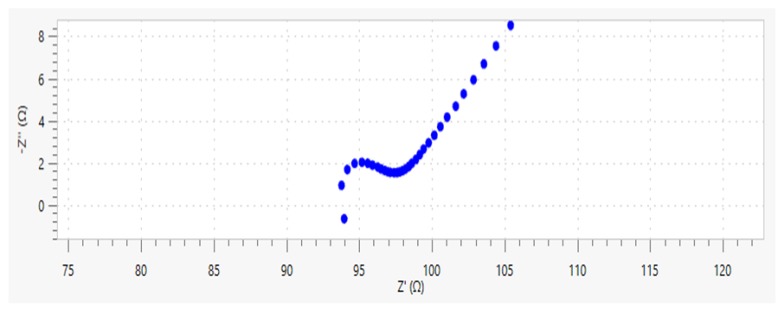
Typical Nyquist diagram of ionically conductive mortar.

The Nyquist plot of the quasi-Randles model is shown in Figure 7 [31]. The Nyquist plot contains two parts: the semicircle (circular arc) in the high frequency region which is controlled by dynamics and the oblique line in the low frequency region which is controlled by diffusion. The intercept of curve with real axis $Z^{\prime }$ in high frequency region is solution resistance $R_{S}$, and the diameter of the semicircle is the charge-transfer resistance $R_{P}$, also known as the interface reaction resistance. Figure 8 shows the typical Nyquist plot of ionically conductive mortar. It can be found that the Nyquist plot of ionically conductive mortar is also composed of the semicircle in the high frequency region and diffusion line in the low frequency region, which conforms to the quasi-Randles model. Thus, the quasi-Randles model was adopted in the following EIS research of the ionically conductive mortar.

### 4.2. Test Method 

**Figure 9:**
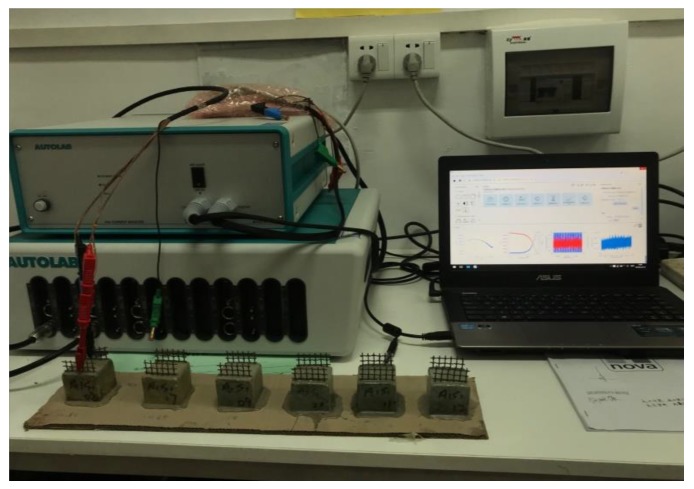
EIS test.

The electrochemical impedance spectrum of ionically conductive mortar was also measured by Autolab M204 electrochemical workstation. During the test, the electrode of the mortar specimen was tightly connected with the measuring plug of the electrochemical workstation, as shown in Figure 9. The parameters of EIS were set as follows: frequency range was 10–1 Hz ~ 106 Hz; the frequency distribution mode was set as points per decade; the frequency point was 10 per decade; the amplitude of the ac signal was 10 mV; the disturbance signal type was single sine wave. FRA (frequency response analyze) tests were performed internally by PGSTAT 302N, total test time was expected to be about 220 s. The resistivity of the mortar was measured by voltammetry (two electrode method) immediately after the EIS test. The method of resistivity test can be found in reference [6]. EIS test will not damage the specimen or change the microstructure of the specimen. Therefore, it can be assumed that the resistivity and EIS of specimen was tested in the same condition, and the results of two test can be compared.

### 4.3. EIS of Ionically Conductive Mortar with Different Porosity

**Figure 10:**
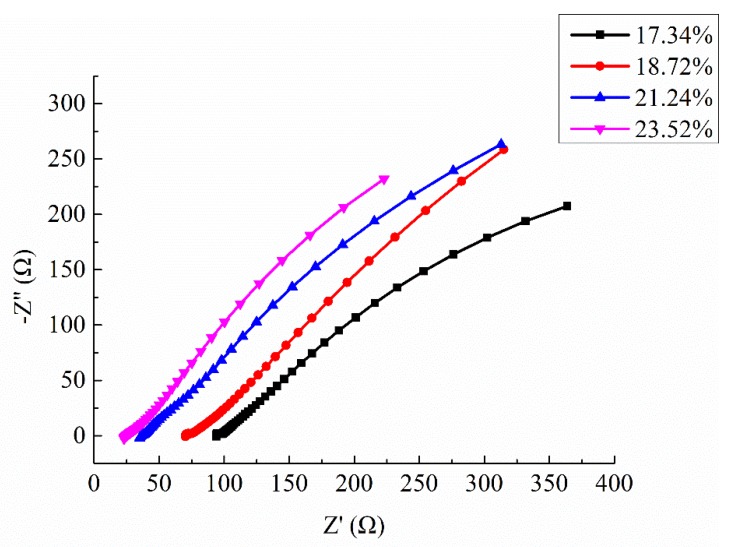
Nyquist diagram of different porosity mortar specimens.

Four samples were selected in this test, with porosity of 17.34%, 18.72%, 21.24%, and 23.52%, respectively. The permeated electrolyte solution was 10% CaCl_2_ solution (mass ratio), and the Nyquist plots of specimens are shown in Figure 10. It is worth noting that the semicircle in the high frequency region just appears in the very beginning, with a small diameter, and then it starts to gradually diffuse. Therefore, the small semicircle in high frequency of Nyquist plots at this test is not very obvious.

As can be seen from Figure 10, the shape and trend of the four curves are very similar which proves the stability of the test. However, the intercept between the extension line of semicircle in high frequency and the real axis $Z^{\prime }$ decreased with the increasing of porosity. As described in Section 4.1, the intercept of curve with real axis $Z^{\prime }$ in high frequency region is solution resistance $R_{S}$. Thus, the results of test also means that the $R_{S}$ decreased with the increasing of porosity. It has been proved by the permeating test that the penetration efficiency was improved with the increasing porosity. The electrolyte solution inside the mortar also increased with the increasing porosity. These are the reasons $R_{S}$ decreased with the increasing porosity. The equivalent circuit of ionically conductive mortar also proves this rule. 

**Figure 11:**
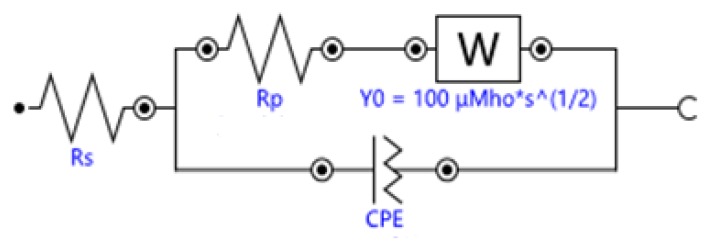
Equivalent circuit diagrams of ionically conductive mortar.

According to the analysis in Section 4.1, the basic equivalent circuit model of ionically conductive mortar is quasi-Randles model. Nova 2.1.3, the software installed in the electrochemical workstation of Autolab, was used to simulate the Nyquist plots of the above four samples. The equivalent circuit diagrams of ionically conductive mortar is shown in Figure 11. The parameters of equivalent circuit and resistivity of specimens with different porosity are shown in Table 8. 

As can be seen from Table 8 and Figure 11, as the porosity of the mortar specimen increases, the solution resistance $R_{S}$ and charge transfer resistance $R_{P}$ both decrease, while the interface capacitance CPE increases.

As mentioned before, the impedance of ionically conductive mortar is almost entirely determined by its pore structure characteristics and electrolyte solution in the pores. With the increase of the porosity of the specimen, more electrolyte solution penetrates into the interior of the specimen, the number of ions in the pore solution also gradually increases, and the solution conductivity is enhanced by more ions moving in the direction of the electric field. It was proved that the solution resistance $R_{S}$ depends on the concentration of the electrolyte in the liquid phase and the solid phase area [25]. Thus, the change of $R_{S}$ can reflect the change of ion concentration of the solution inside pore and porosity, and the porosity occupies a dominant position in the two factors, and the size of the resistance is inversely proportional to the specimen porosity [35]. Therefore, the porosity of ionic conductive mortar decreases with the increase of porosity.

In fact, when the porosity of the specimens increases the size of internal pore also increases, so as to improve the inter-connectivity inside the mortar. The movement of ions inside the mortar become easier, which also means the transfer of electric charge is easier, the solid–liquid interface reaction is also increasingly fierce. Therefore, with the increase of specimen porosity, the charge-transfer resistance (interface reaction resistance) $R_{P}$ decreases. At the same time, greater porosity also means more electrolyte solution in the hole, which leads to the increase of charge at the solid–liquid interface and hence the increase of solid–liquid interface capacitance CPE.

**Figure 12:**
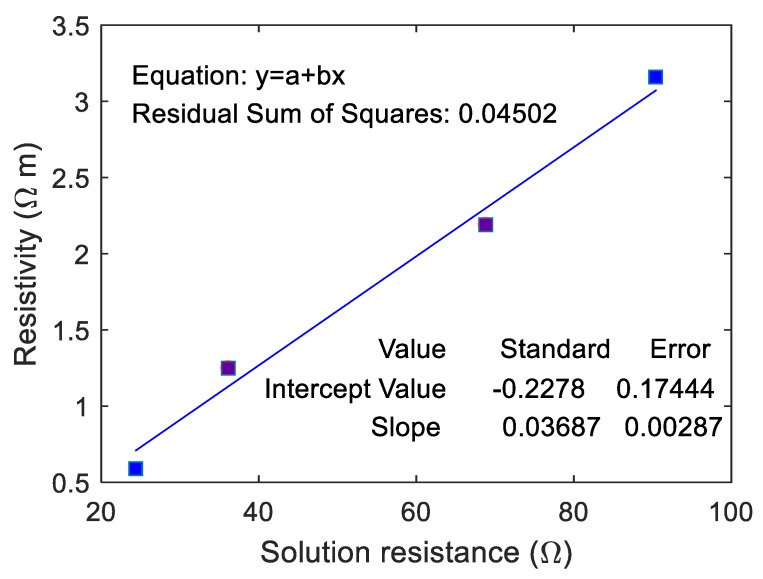
Correlation between resistivity and solution resistance of mortar specimen.

The correlation between the resistivity of ionically conductive mortar specimen and $R_{S}$ was fitted, and the results are shown in Figure 12. As can be seen from the figure, the resistivity of ionically conductive mortar and $R_{S}$ are linearly correlated, and the correlation coefficient reaches 0.982. The fitting equation of the line is y=0.037x−0.227. The real resistivity of the mortar specimen increases with the increase of $R_{S}$. $R_{S}$ means the resistance of solution inside the pore and pore area. Thus, when the solution resistance $R_{S}$ increases, it means that the resistivity of electrolyte solution increases and pore area decreases, consequently causing increase in the resistivity of the specimen. On the other hand, the linear correlation between the real resistivity of ionically conductive mortar specimen and $R_{S}$ also proved the validity and effectiveness of the equivalent circuit. 

### 4.4. EIS of Ionically Conductive Mortar with Different Concentration of Electrolyte Solution

**Figure 13:**
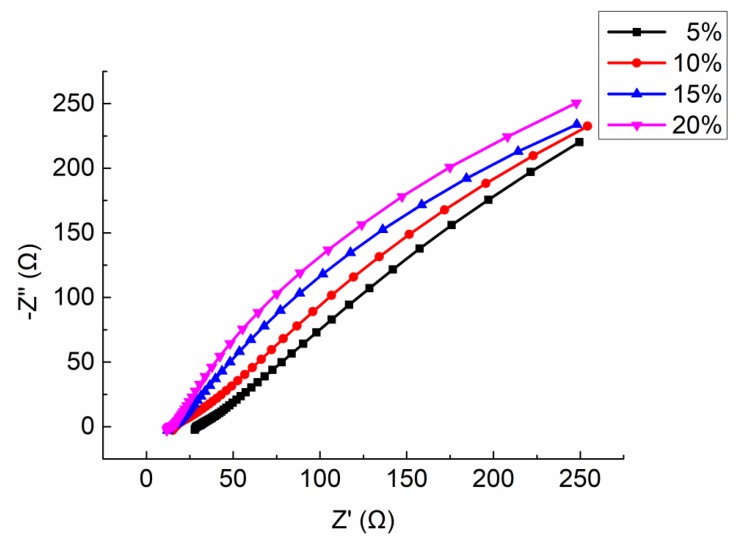
Integrated Nyquist plot of the specimens with different mass ratio of electrolyte solution.

Four mortar specimens (porosity 23.3 ~ 23.7%) made in the same time and with the same formula were selected. The electrolyte solution was CaCl_2_ with mass ratio 5%, 10%, 15%, and 20%, respectively. The Nyquist plots are shown in Figure 13. The equivalent circuit diagrams are shown in Figure 11. The parameters of equivalent circuit of specimen with different electrolyte solution concentration are shown in Table 9 (including the measured resistivity of specimens).

It can be seen from the Figure 13 that the shape and trend of the four curves are very similar which proves the stability of the test. However, the intercept between the extension line of semicircle in high frequency and the real axis $Z^{\prime }$ decrease with the increasing of the mass ratio of electrolyte solution. Combining the parameters shown in Table 9, with the increase of the electrolyte solution mass ratio, the solution resistance $R_{S}$ and the charge transfer resistance $R_{P}$ both decrease, but the interface capacitance CPE increases.

As mentioned before, the resistivity of ionically conductive mortar is almost entirely determined by its pore structure characteristics and electrolyte solution inside pores. In this test, all the specimens were manufactured in the same time with the same mix ratio, thus, the porosity and internal pore structure characteristics were similar. Under this condition, the electrolyte solution inside the pores become the main factor affecting the resistivity of the specimen. Meanwhile, the solution resistance $R_{S}$ is determined by the concentration of electrolyte solution in the specimen and the solid phase area [34]. When the porosity and pore structure characteristics are similar, the value of $R_{S}$ also is determined by the concentration of electrolyte solution. It has been proved that $R_{S}$ is inversely proportional to the total concentration of ions inside pores [35]. This is because the conductivity of the electrolyte solution is determined by the concentration of free moving ions in the solution and the charge they carry. The higher the concentration of free moving ions is, the more the charge the ions carry, the stronger the conductivity of the solution will be. It is worth noting that when the concentration of solution is lower than a certain level, the quantity of free ions dominates the conductivity of electrolyte solution, and the conductivity of solution is enhanced because of the increasing number of free ions. When the concentration of solution exceeds the certain level, the interaction force between ions increases, which declines the movement velocity of free ions in the solution. At this circumstance, the conductivity of solution decreases with the increase of concentration. Therefore, for electrolyte solution, there is a threshold value of concentration of electrolyte solution to its conductivity. It can be found in Table 9 when the mass ratio of electrolyte solution increased from 5% to 10%, solution resistance $R_{S}$ dropped from 29.13 Ω to 16.73 Ω, which proves the conductivity of electrolyte solution would increase when the concentration increases until reaching the threshold value. When the mass ratio increased from 10% to 15%, the change range of $R_{S}$ became flat gradually. When the mass fraction increased from 15% to 20%,$R_{S}$ only decreased from 13.82 Ω to 13.41 Ω. This is because that 20% mass ratio approaches the concentration of CaCl_2_ solution. It can be predicted that if the solution concentration increased continually, the conductivity of CaCl_2_ solution would decrease once the mass ratio exceed the threshold value, which also declines the conductivity of ionically conductive mortar.

However, the interfacial capacitance CPE increases with the increase of concentration of electrolyte solution. This is because with the increase of the concentration of electrolyte solution, the number of free ions increases, and the charge they carry also increases; these conditions make the solid–liquid interface reaction become fierce, consequently leading to the increase of the charge at the solid–liquid interface and hence the increase of solid-liquid interface capacitance CPE.

**Figure 14:**
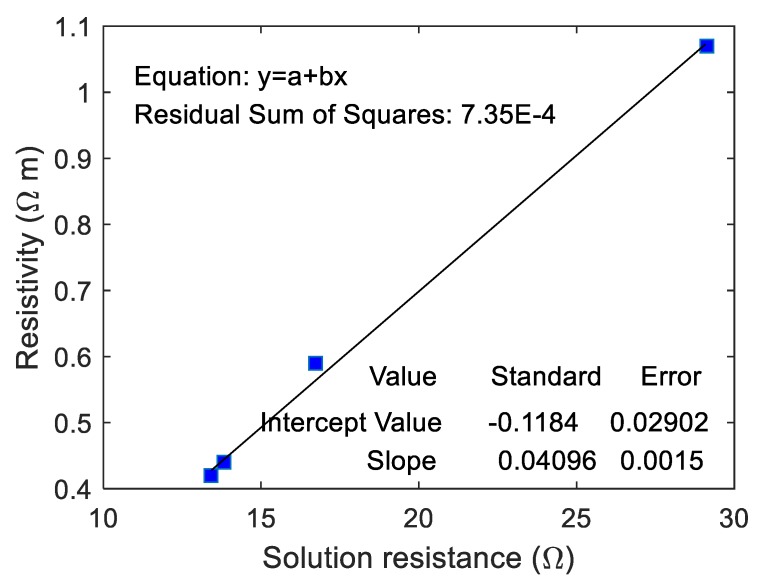
Correlation between resistivity and solution resistance of mortar specimens with different mass ratio of electrolyte solution.

The correlation between the real resistivity of ionically conductive mortar specimens and $R_{S}$ of the equivalent circuit was fitted, and the results are shown in Figure 14. As can be seen from the figure, the resistivity of ionically conductive mortar and $R_{S}$ are linearly correlated, and the correlation coefficient reaches 0.996. The fitting equation of the line is y=0.041x−0.118. The real resistivity of the mortar specimen increases with the increase of $R_{S}$. The validity and effectiveness of equivalent circuit calculated by EIS has been proved again by this linear equation. 

### 4.5. EIS of Ionically Conductive Mortar with Different Solute

**Figure 15:**
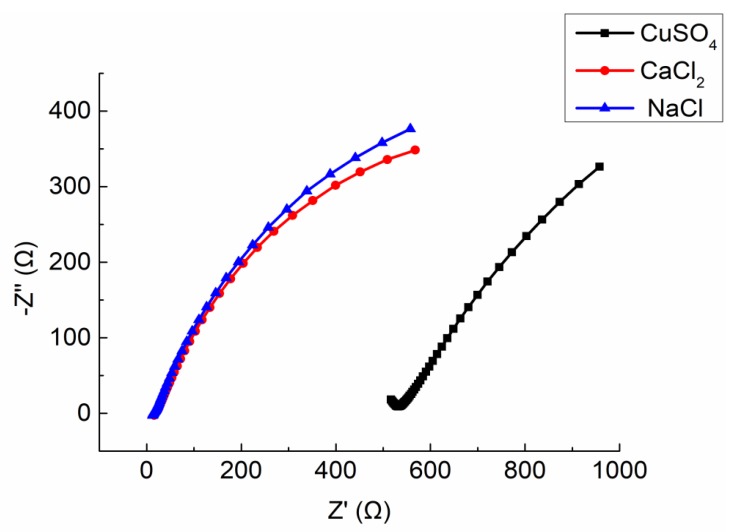
Integrated Nyquist plot of the specimens with different solute.

Three mortar specimens (porosity 23.3 ~ 23.7%) made in the same time and with the same mix ratio were selected. The solutes used to prepare electrolyte solution were CaCl_2_, CuSO_4_, and NaCl, respectively. The mass ratio of three electrolyte solution is 10%. The Nyquist plots are shown in Figure 15. The equivalent circuit diagram of ionically conductive mortar is shown in Figure 11. The parameters of equivalent circuits with different electrolyte solution are shown in Table 10 (including the measured resistivity of specimens).

It can be seen from the Figure 15 that the Nyquist plots of the specimens with CaCl_2_ and NaCl solutions are similar, while the specimen with CuSO_4_ solution are much different from the other two. The intercept between the extension line of semicircle in high frequency and the real axis $Z^{\prime }$ of the specimen with CuSO_4_ solution is much larger than that of the specimen permeated by Cl^-^ solution. As shown in Table 10, the different types of electrolyte solutions will lead to the differences in the solution resistance $R_{S}$, charge transfer resistance $R_{P}$, and interface capacitance CPE of equivalent circuit. Among them, the relationship between $R_{S}$ and $R_{P}$ is CuSO_4_>CaCl_2_>NaCl, while the CPE shows the opposite trend, which is NaCl > CaCl_2_ > CuSO_4_.

As described before, when the specimens were manufactured in the same time with the same mix ratio, the main factor affecting the resistivity of the specimen is the electrolyte solution inside pores. The conductivity of electrolyte solution is determined by the concentration and movement velocity of free ions in the solution. The mass ratio cannot accurately represent the concentration of ions in the solution since the type of electrolyte solutions in this section is different. Therefore, it is necessary to convert the mass fraction into the molarity of solute substances, so as to compare the concentration of different electrolyte solutions. The formula for the molarity of solute substances can be calculated as Equation (1) [36]:

(1) $$c=\frac{1000\times \rho \cdot w}{M}$$

where, ρ is the density of electrolyte solution, which can be tested after solution being prepared, *w* is the mass ratio of the solution, and *M* is the molar mass of the solute substance. For the three solutions selected in this section, molarity of solute substances was calculated by Equation (1), the results are shown in Table 11. 

As shown in Table 11, the molarity of the three solutes is: NaCl > CaCl_2_ > CuSO_4_, which means the solution of NaCl has largest amounts of free ions. Therefore, solution of NaCl has the strongest conductivity, while CuSO_4_ solution is the weakest. As test results shown in Table 10, $R_{S}$ of specimen with NaCl solution is the smallest, only 14.44 Ω, the next is 17.77 Ω of the specimen with CaCl_2_, and the $R_{S}$ of specimen with CuSO_4_ solution reached 411.96 Ω, far higher than the front two specimens. 

The $R_{P}$ and CPE of specimen also were influenced by the concentration of free ions in the solution. The $R_{P}$ increases with the increase of the concentration of free ions in the solution, while the CPE decreases. The reason of this rule can be found in Section 4.4. In this test, the order of concentration of free ions is NaCl > CaCl_2_ >CuSO_4_, the order of the $R_{P}$ is CuSO_4_ > CaCl_2_ > NaCl, and the order of the CPE is NaCl > CaCl_2_ > CuSO_4_, all of the test results conform to the rule above. 

**Figure 16:**
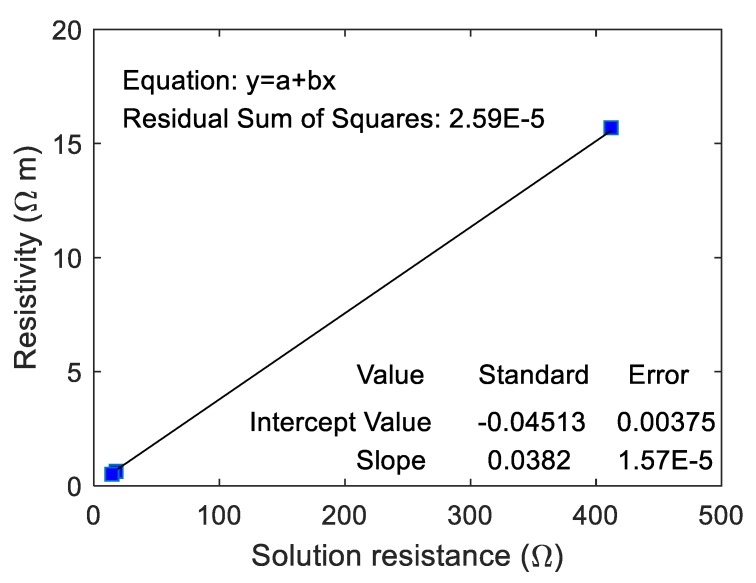
Correlation between resistivity and $R_{S}$ of specimens with different solute.

The correlation between the real resistivity of ionically conductive mortar specimen and the $R_{S}$ was fitted, and the results are shown in Figure 16. In this case, the real resistivity of ionically conductive mortar specimen and $R_{S}$ are also linearly correlated. The correlation coefficient even reaches 1, and the equation of the fitting line is y=0.038x−0.045. It shows that the equivalent circuit according to the EIS tested by electrochemical workstation can effectively reflect the true resistance of the ionically conductive mortar.

## 5. Conclusions

The electrochemical workstation was used in this paper to test the open circuit potential of for ionically conductive mortar during the permeating process. Further, the Nyquist plots of ionically conductive mortar were also tested by electrochemical workstation. Based on this, the equivalent circuit of ionically conductive mortar was simulated, and the correlation of real resistivity of specimen and parameter $R_{S}$ of equivalent circuit was fitted to prove the effectiveness of the equivalent circuit. The main conclusions are as follows:

(1) Open-circuit potential during permeating process can effectively and truly reflect the influence of different porosity and different mass ratios of the electrolyte solution on the permeability of ionic conductive mortar. The experimental results showed that the higher the porosity, the easier the electrolyte solution penetrates, the larger the slope of the measured open circuit potential curve, consequently the smaller the real resistivity of the ionically conductive mortar. For the mass ratio of electrolyte solution, there is an optimal value to get the best permeability of ionically conductive mortar, and this value would change if solute changes. 

(2) Quasi-Randles model can be used as the equivalent circuit model of ionically conductive mortar. Quasi-Randles model consists of solution resistance $R_{S}$, charge-transfer resistance $R_{P}$, solid-liquid interface capacitance CPE, and diffusion impedance $Z_{W}$. The value of those parameters were mainly influenced by the concentration and movement velocity of free ions inside the mortar. With the increase of the porosity of the mortar specimen, the concentration of free ions highly increased, therefore, the $R_{S}$ and the $R_{P}$ decreased, but the CPE increased because of the increasing charge carried by the free ions. For the mass ratio of electrolyte solution, there is also a threshold value on the conductivity of electrolyte solution because the movement velocity of the free ions reduces if the concentration of free ions exceeds a certain level. Therefore, the $R_{S}$ and $R_{P}$ increased with the mass ratio of electrolyte solution in the certain section, but this rule would be broken once the mass ratio exceeds the threshold value. For the electrolyte solution with different solute, the concentration of free ions cannot be considered only by mass ratio but also by solute molarity. The concentration of free ions increased with the increase of solute molarity. As mentioned before, the conductivity of electrolyte solution does not increase with the increase of concentration of free ions continuously, and there is a threshold value for concentration of ions. This value would change if the solute substance changes. 

(3) The correlation between the parameter $R_{S}$ of equivalent circuit and real resistivity of ionically conductive mortar specimens were fitted under condition of different porosity, different mass ratio of electrolyte solution and different solute, respectively. There is a strong linear correlation between the $R_{S}$ and the real resistivity in the three conditions mentioned above. This phenomenon shows that the equivalent circuit based on the EIS tested by electrochemical station can truly and effectively reflect the real circuit inside the ionically conductive mortar.

The EIS test results and corresponding equivalent circuit in this paper were obtained from limited number of specimens. The conclusion needs to be verified by more specimens in further study. 

## Figures and Tables

**Table 1:** Material properties.

Materials	Properties of Materials
Cement	Portland cement PO325, ignition loss 2.28%, initial setting time ≥ 45 min, chemical composites are shown in Table 2
Sand	Ordinary river sand (SiO_2_), 50% of the total mass particle size ≤ 0.25 mm, the average particle size: 0.25–0.5 mm
Water	Ordinary tap water, composites are shown in Table 3
Copper electrode	Diameter 1 mm, aperture 5 mm × 5 mm, processed into a size of 40 mm × 65 mm sheet as the electrode
Aluminum powder	used as 99.5% purity, diameter 60 ~ 75 μm
Triterpene saponin air entraining admixture SJ-2	Light yellow powder; content of natural triterpene saponin≥63%
CaCl_2_	Electrolyte solution for immersing the specimens; Analytical reagent; content ≥ 99%
CuSO_4_	Electrolyte solution for immersing the specimens; Analytical reagent; content ≥ 99%
NaCl	Electrolyte solution for immersing the specimens; Analytical reagent; content ≥ 99%

Note: 1 MPa = 145 psi; 1 in. = 25.4 mm.

**Table 2:** Cement chemical composite.

Composites	CaO	SiO_2_	Al_2_O_3_	Fe_2_O_3_	MgO	K_2_O	Na_2_O	SO_3_	Cl^-^
Content (wt.%)	62.17	21.84	6.56	4.15	2.23	0.34	0.41	2.26	0.013

**Table 3:** Water composite (PH = 7.56).

Composites	Al	Fe	Mn	Cu	Zn	Cl^-^	SO_4_^-^	NO_3_^-^	As	Cr(VI)	Se
Content (mg/L)	0.03	<0.05	<0.05	<0.05	<0.05	8.4	29.9	0.62	<0.001	<0.004	<0.001

**Table 4:** The specimen codes for open-circuit potential test.

The Specimens with Different Porosity		The Specimens with Different Electrolyte Solution Concentration	
Code	Porosity	Code	electrolyte solution concentration
OP-1	18.72%	OC-1	5%
OP-2	21.65%	OC-2	10%
OP-3	23.71%	OC-3	15%
OP-4	25.87%	OC-4	20%

**Table 5:** The specimen codes for electrochemical impedance spectroscopy (EIS) test.

The Specimens with Different Porosity		The Specimens with Different Electrolyte Solution Concentration		The Specimens with Different Solute	
Code	Porosity	Code	electrolyte solution concentration	Code	Solute
EP-1	17.34%	EC-1	5%	ES-1	CuSO_4_
EP-2	18.72%	EC-2	10%	ES-2	CaCl_2_
EP-3	21.24%	EC-3	15%	ES-3	NaCl
EP-4	23.52%	EC-4	20%		

**Table 6:** Change of open circuit potential with time of different porosity specimens.

Code	Porosity	Open Circuit Potential (V)							Resistivity (Ω⋅m)
		0 min	3 min	6 min	9 min	12 min	15 min	∆V	
OP-1	18.72%	0.0299	0.0141	0.0016	−0.0054	−0.0082	−0.0133	0.0432	38.27
OP-2	21.65%	0.0692	−0.0026	−0.0116	−0.0161	−0.0218	−0.0229	0.0921	8.04
OP-3	23.71%	0.0324	−0.0328	−0.0250	−0.0368	−0.0491	−0.0645	0.0969	7.30
OP-4	25.87%	0.0506	−0.0450	−0.0537	−0.0576	−0.0656	−0.0734	0.1240	5.39

1. ∆V=V(15min)−V(0min); 2. The resistivity in this paper was test by two electrode methods under AC (alternative current) 10V [6,7,8].

**Table 7:** Change of open circuit potential of specimens with different mass ratio electrolyte solution.

Code	Mass Ratio of Electrolyte Solution	Open Circuit Potential (V)							Resistivity (Ω⋅m)
		0 min	3 min	6 min	9 min	12 min	15 min	∆V	
OC-1	5%	−0.0033	−0.0048	−0.0085	−0.0157	−0.0228	−0.0270	0.0237	1.07
OC-2	10%	−0.0026	−0.0402	−0.0581	−0.0726	−0.0808	−0.0816	0.0790	0.59
OC-3	15%	−0.0058	−0.0486	−0.0675	−0.0863	−0.1015	−0.1002	0.0944	0.44
OC-4	20%	−0.0013	−0.0966	−0.1100	−0.1175	−0.1168	−0.1141	0.1129	0.42

**Table 8:** Equivalent circuit parameters and resistivity of specimens with different porosity.

Code	Porosity	$R_{S}\;(\Omega )$	$R_{P}\;(\Omega )$	CPE (F)	ρ (Ω⋅m)
EP-1	17.34%	90.39	9.03	3.83 × 10^−8^	3.16
EP-2	18.72%	68.82	8.72	9.95 × 10^−8^	2.19
EP-3	21.24%	36.13	8.24	4.18 × 10^−6^	1.25
EP-4	23.52%	24.36	7.41	6.71 × 10^−6^	0.59

ρ means the resistivity of specimen which was measured by voltammetry (two electrode) [6].

**Table 9:** Parameters of equivalent circuit and resistivity of the specimens with different mass ratio of electrolyte solution.

Code	Mass Ratio of Electrolyte Solution	$R_{S}\;(\Omega )$	$R_{P}\;(\Omega )$	CPE (F)	ρ (Ω⋅m)
EC-1	5%	29.13	17.92	9.42 × 10^−6^	1.07
EC-2	10%	16.73	15.59	1.06 × 10^−5^	0.59
EC-3	15%	13.82	12.94	7.35 × 10^−5^	0.44
EC-4	20%	13.41	4.86	9.40 × 10^−5^	0.42

**Table 10:** Parameters of equivalent circuit and resistivity of the specimens with different solute.

Code	Type of Electrolyte Solution	$R_{S}\;(\Omega )$	$R_{P}\;(\Omega )$	CPE (F)	ρ (Ω⋅m)
ES-1	CuSO_4_	411.96	122.00	1.30 × 10^−8^	15.69
ES-2	CaCl_2_	17.77	13.82	5.39 × 10^−5^	0.63
ES-3	NaCl	14.44	13.70	5.98 × 10^−5^	0.51

**Table 11:** Molarity of different solute.

Type of Electrolyte Solution	ρ (g/mL)	w (%)	M (g/mol)	c (mol/L)
CuSO_4_	1.078	10%	160	0.674
CaCl_2_	1.086	10%	111	0.978
NaCl	1.069	10%	58.5	1.827
